# Supplementary material for: Studies on Improving the Efficiency of Somatic Embryogenesis in Grapevine (Vitis vinifera L.) and Optimising Ethyl Methanesulfonate Treatment for Mutation Induction
Source: Plants (Basel). 2023 Dec 11;12(24):4126. doi: 10.3390/plants12244126 (PMC10748286; doi:10.3390/plants12244126)
Supplement: Supplementary file 1 [file plants-12-04126-s001.zip › plants-2727565-supplementary.pdf]

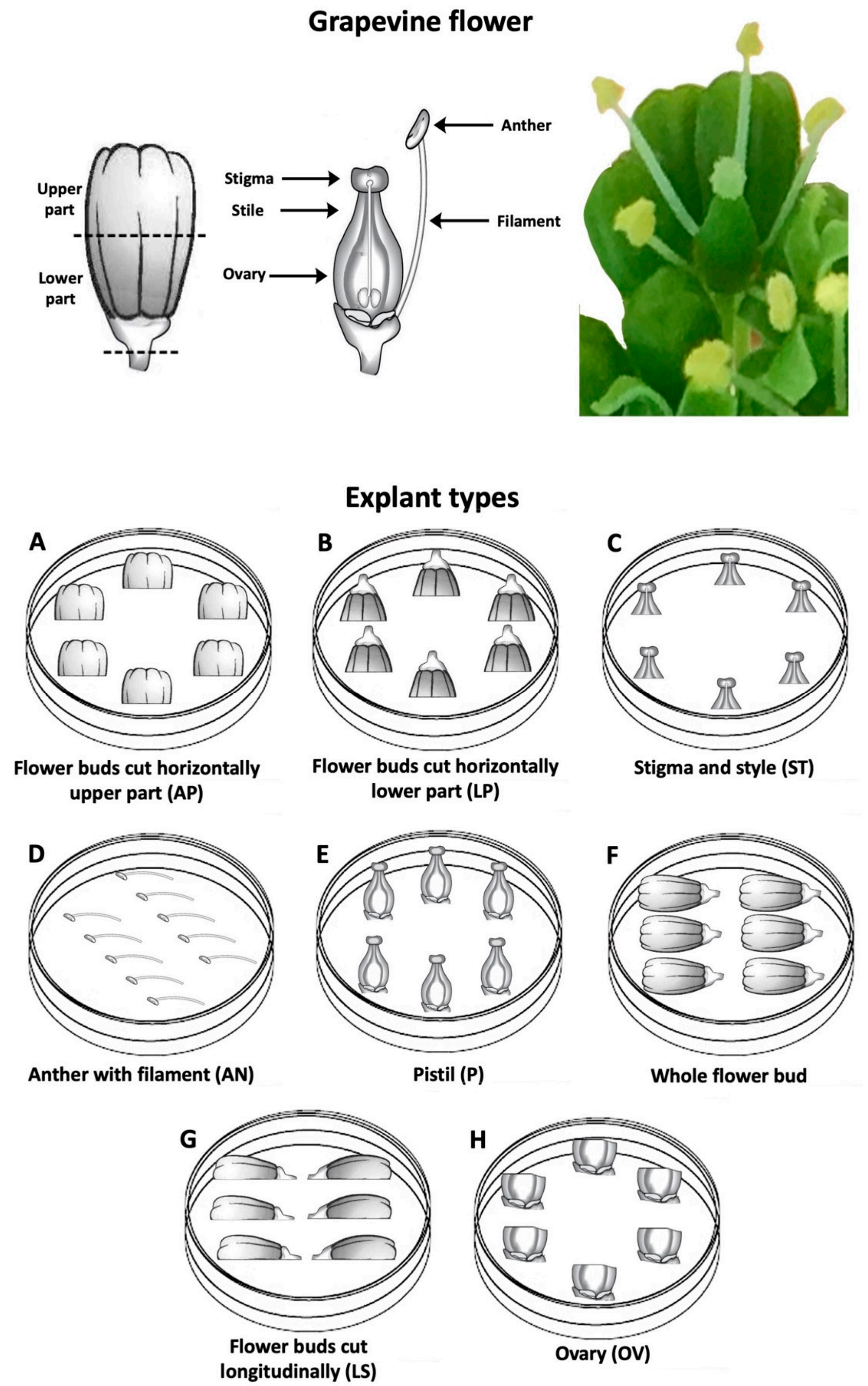

Figure S1. Flower parts and explants tested. Explant types: (**A**) Flower buds cut horizontally upper part (AP); (**B**) Flower buds cut horizontally lower part (LP); (**C**) Stigma and style (ST); (**D**) Anther with filament (AN); (**E**) pistil (P); (**F**) Whole flower bud; (**G**) Flower buds cut longitudinally (LS); (**H**) Ovary (OV).

Table S1. Results of statistical analysis during somatic embryogenesis in grapevine using a binomial generalised linear model in three experiments involving two media, 3-5 explant types in three white grape cultivars.

**Analysis of Deviance**	**DF**	**Callused/Cultured**		**Embryogenic/Cultured**		**Embryogenic/Callused**	
**Experiment 1**		Dev	*p*	Dev	*p*	Dev	*p*
Cultivar	1	5.0	0.025	0.5	0.483	0.009	0.925
Explant	2	65.7	<0.001	3.6	0.164	4.391	0.111
Medium	1	137.5	<0.001	6.4	0.011	1.389	0.239
Cultivar.Explant	2	24.0	<0.001	0.9	0.640	0.542	0.763
Cultivar.Medium	1	3.8	0.050	0.2	0.629	3.757	0.053
Explant.Medium	2	24.6	<0.001	0.7	0.700	0.807	0.668
Cultivar.Explant. Medium	2	9.0	0.011	2.4	0.298	0.205	0.651[a]
Residual	24	29.7		22.8		27.776 [a]	
**Experiment 2**							
Cultivar	2	5.0	0.082	1.7	0.425	0.9	0.651
Explant	4	142.1	<0.001	20.1	<0.001	1.5	0.824
Medium	1	631.1	<0.001	45.1	<0.001	11.0	<0.001
Cultivar.Explant	8	14.2	0.078	5.6	0.692	4.5	0.809
Cultivar.Medium	2	0.1	0.960	0.0	0.980	1.0	0.595
Explant.Medium	4	78.7	<0.001	14.2	0.007	0.2	0.984[b]
Cultivar.Explant.Medium	8	9.3	0.319	4.6	0.802	4.2	0.517[b]
Residual	70	77.4		63.9		53.0 [b]	
**Experiment 3**							
Cultivar	2	10.5	0.072	2.7	0.264	2.6	0.278
Explant_	7	319.2	<0.001	8.8	0.032	14.9	0.002
Explant_maturity	1	0.2	0.756	32.0	<0.001	30.4	<0.001
Medium	1	2627	<0.001	0.1	0.736	10.2	0.001
Cultivar.Explant_Typ	6	95.7	<0.001	19.6	0.003	15.1	0.020
Cultivar.Explant_Mat	2	0.5	0.866	1.9	0.396	2.7	0.261
Explant_Typ.Explant_Mat	3	6.9	0.315	4.7	0.196	2.5	0.481
Cultivar.Medium	2	11.5	0.057	7.4	0.025	3.6	0.161
Explant_Typ.Medium	3	73.1	<0.001	2.6	0.451	0.7	0.866
Explant_Mat.Medium	1	28.3	<0.001	2.6	0.108	1.4	0.242
Cultivar.Explant_Typ. Explant_Mat	6	43.2	0.003	2.5	0.874	0.7	0.995
Cultivar.Explant_Typ. Medium	6	34.2	0.013	4.1	0.660	3.3	0.770
Cultivar.Explant_Mat. Medium	2	22.6	0.005	15.3	<.001	18.4	<0.001
Explant_Mat.Explant_Typ. Medium	3	14.0	0.073	2.0	0.579	0.1	0.988
Cultivar.Explant_Typ Explant_Mat.Medium .	6	8.1	0.641	8.6	0.200	7.5	0.023[c]
Residual	55	104.8		60.1		58.7[c]	

[a] because there were no calluses in some treatments, the Cultivar.Explant.Medium effect has 1 df and the residual 24 df.

[b] because there were no calluses in some treatments, the Explant.Medium effect has 3 df, Cultivar.Explant.Medium has 5 df and the residual 54 df.

[c] because there were no calluses in some treatments, the Cultivar.Explant_Typ.Explant_Mat.Medium effect has 2 df and the residual 46 df.

Table S2. The proportion of explants callusing and embryogenic from cultured explants, and the proportion of callused explants producing somatic embryos in Experiment 1.

Estimated Proportions	Callused/Cultured		Embryogenic/Cultured		Embryogenic/Callused	
	**2337**	**2339**	**2337**	**2339**	**2337**	**2339**
**Riesling LP**						
**ST**	11%	71%	4%	10%	33%	14%
**UP**	3%	20%	2%	5%	50%	25%
**Sauvignon blanc**	8%	70%	3%	5%	40%	7%
**LP ST**						
**UP**	13%	77%	2%	10%	13%	13%
	15%	7%	3%	3%	22%	50%
**Approx LSD**	0%	22%	0%	3%	*	15%
	Average 13%		Average 7%		Average 47%	
	Range 5% to 18%		Range 3% to 12%		Range 13% to 90%	

Table S3. The proportion of explants callusing and embryogenic from cultured explants, and the proportion of callused explants producing somatic embryos in Experiment 2. As there was no variety effect, the overall means for the three cultivars are presented

Estimated Proportions	Callused/Cultured		Embryogenic/Cultured		Embryogenic/Callused	
	2337	2339	2337	2339	2337	2339
**Anther**	0%	93%	0%	18%	*	19%
**LS**	14%	69%	4%	10%	34%	15%
**Pistil**	8%	31%	3%	5%	38%	17%
**TS**	12%	86%	5%	16%	37%	19%
**Whole flower**	5%	33%	1%	7%	31%	21%
**Approx LSD**	Average 7%		Average 5%		Average 22%	
	Range 3% to 10%		Range 1% to 10%		Range 6% to 40%	

Table S4. The proportion of explants callusing and embryogenic from cultured explants, and the proportion of callused explants producing somatic embryos in Experiment 3.

**Estimated Proportions**	**Callused/Cultured**		**Embryogenic/Cultured**		**Embryogenic/Callused**	
	2337	2339	2337	2339	2337	2339
**Chardonnay**						
**AN-I**	15%	95%	0%	0%	0%	0%
**AN-M**	0%	100%	0%	0%	*	0%
**OV-I**	48%	51%	23%	0%	47%	0%
**OV-M**	38%	65%	0%	5%	0%	8%
**P-I**	33%	42%	15%	10%	46%	23%
**P-M**	20%	43%	0%	3%	0%	6%
**ST-I**	18%	31%	10%	3%	57%	9%
**ST-M**	25%	48%	0%	3%	0%	5%
**Riesling**						
**AN-I**	55%	95%	3%	5%	5%	5%
**AN-M**	54%	100%	1%	5%	2%	5%
**OV-I**	23%	78%	3%	13%	11%	16%
**OV-M**	15%	80%	2%	0%	11%	0%
**P-I**	8%	13%	3%	3%	33%	20%
**P-M**	0%	38%	0%	3%	*	7%
**ST-I**	3%	20%	0%	3%	0%	13%
**ST-M**	10%	10%	0%	0%	0%	0%
**Sauv Blanc**						
**AN-I**	20%	58%	3%	8%	13%	13%
**AN-M**	28%	100%	8%	0%	27%	0%
**OV-I**	60%	60%	13%	5%	21%	8%
**OV-M**	20%	95%	0%	3%	0%	3%
**P-I**	23%	28%	5%	3%	22%	9%
**P-M**	0%	5%	0%	0%	*	0%
**ST-I**	3%	18%	0%	5%	0%	27%
**ST-M**	0%	8%	0%	0%	*	0%
**Approx LSD**	Average 22%<br>Range 1% to 34%		Average 7%<br>Range 1% to 17%		Average 21%<br>Range 1% to 67%	
